# Enzymatic Study of Linoleic and Alpha-Linolenic Acids Biohydrogenation by Chloramphenicol-Treated Mixed Rumen Bacterial Species

**DOI:** 10.3389/fmicb.2018.01452

**Published:** 2018-07-03

**Authors:** Annabelle Meynadier, Asma Zened, Yves Farizon, Marie-Luce Chemit, Francis Enjalbert

**Affiliations:** GenPhySE, INRA, ENVT, Université de Toulouse, Toulouse, France

**Keywords:** rumen, bacteria, unsaturated fatty acids, biohydrogenation, enzymes

## Abstract

In the rumen, dietary polyunsaturated fatty acids (PUFA) are reduced by a multistage reaction called biohydrogenation (BH). BH leads to a high proportion of saturated fat in ruminant products, but also products some potential bioactive intermediates like conjugated linoleic and linolenic acids. BH is composed of two kinds of reactions: first an isomerization of PUFA followed by reductions (two for linoleic acid, C18:2n-6; three for α-linolenic acid, C18:3n-3). There is little knowledge about BH enzymes as BH bacterial species are the subject of a lot of studies. Nevertheless, both aspects must be explored to control BH and enhance the fatty acids profile of ruminant products. In the present study, an alternative approach was developed to study the enzymes produced *in vivo* by mixed ruminal bacteria, using inactivation of bacteria by chloramphenicol, an inhibitor of protein synthesis in prokaryotes, before *in vitro* incubation. To study C18:2n-6 and C18:3n-3 BH several experiments were used: (1) with different incubation durations (0 to 3) to estimate average rates and efficiencies of all BH reactions, and intermediates production; and (2) with different initial quantities of PUFA (0.25 to 2 mg) to estimate Michaelis–Menten enzymatic parameters, K_m_ and V_max_. A last experiment explored the effect of pH buffer and donor cow diet on C18:2n-6 isomerization pathways. Concerning C18:2n-6 BH, this study confirmed the high saturability of its isomerization, the inhibition of both *trans*11 and *trans*10 pathways by a low pH, and the last reduction to stearic acid as the limiting-step. Concerning C18:3n-3, its BH was faster than C18:2n-6, in particular its isomerization (V_max_ = 3.4 vs. 0.6 mM/h, respectively), and the limiting-step was the second reduction to t11-C18:1. Besides, our mixed isomerases had a higher affinity for C18:2n-6 than for C18:3n-3 (K_m_ = 2.0 × 10^-3^ vs. 4.3 × 10^-3^ M, respectively), but due to their high saturability by C18:2n-6, they had a lower efficiency to isomerize C18:2n-6 than C18:3n-3. Chloramphenicol-treated ruminal fluid would be a meaningful method to study the BH enzymes activities.

## Introduction

**FIGURE 1:**
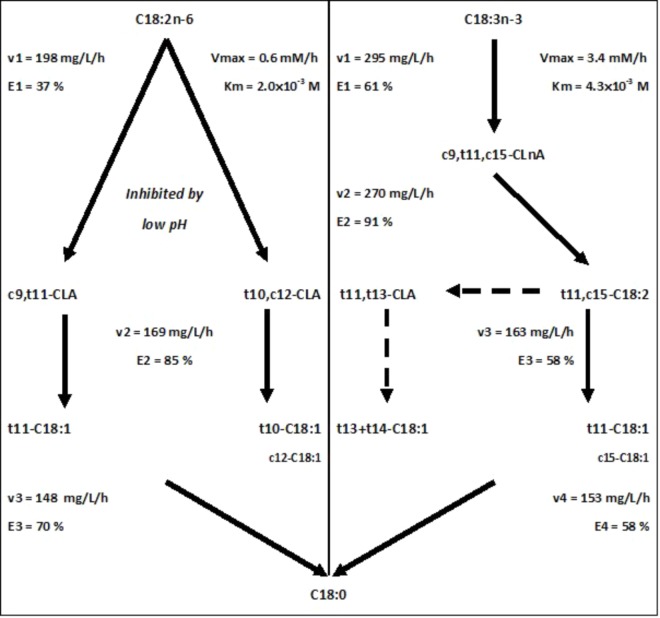
Main pathways of kinetic parameters of biohydrogenation estimated from 1 h incubation studies with 1 mg of C18:2n-6 or C18:3n-3 in an enzymatic solution obtained from a cow’s ruminal content (solid line) and putative origin of t13 isomers (according to Fukuda et al., 2009; dotted line). The rate (v) and the efficiency (E) of the BH reactions were obtained from kinetics of disappearance of C18:2n-6 and C18:3n-3 reported in Experiments 1.1 and 2.1, respectively. K_m_ and V_max_ were estimated from Lineweaver–Burk representation with results of Experiments 1.2 and 2.2, using different concentrations of C18:2n-6 and C18:3n-3, respectively. Experiment 1.3 allowed the conclusion about pH effect.

Ruminal BH (**Figure 1**) corresponds to a bacterial reduction of dietary PUFA and results in the production of intermediate unsaturated FA and finally saturated FA (Enjalbert et al., 2017). The control of BH reactions is of interest because BH directly affects the composition of FA in milk and meat of ruminants.

The linoleic acid (C18:2n-6) BH is divided into three steps (**Figure 1**): an isomerization into CLA followed by a reduction producing mainly *trans*-octadecenoic acids (t-C18:1), and a final reduction producing stearic acid (C18:0). Two main pathways can be involved: a t11 pathway beginning by a Δ12 isomerization and a t10 pathway beginning by a Δ9 isomerization, leading to the formation of intermediates with potential bioactive properties: vaccenic (t11-C18:1) and rumenic (c9,t11-CLA) acids, and t10-C18:1 and t10c12-CLA, respectively (Doreau et al., 2016).

The α-linolenic acid (C18:3n-3) BH is divided into four steps (**Figure 1**): an isomerization into CLnA, then a first reduction producing *trans*-octadecadienoic acids (t-C18:2), which are subsequently reduced to t-C18:1 and a final reduction to C18:0. The main C18:3n-3 BH pathway mainly produces t11,c15-C18:2, t11,t13-CLA, and t11-C18:1 and t13+14-C18:1 (Doreau et al., 2016).

The reactions of BH are catalyzed by bacterial enzymes. *Butyrivibrio fibrisolvens* is the best known ruminal biohydrogenating bacterium, hydrogenating C18:2n-6 and C18:3n-3 by the t11 pathway, and has been used for studying Δ12 isomerase (Kepler and Tove, 1967). *Propionibacterium acnes* has been used for the purification and the study of C18:2n-6 Δ9 isomerase (Deng et al., 2007). However, similar to the diversity of rumen lipases (Privé et al., 2015), other enzymes produced by other bacterial species could be involved in both pathways (Enjalbert et al., 2017), so that, these approaches using pure bacterium could fail to reflect mixed enzymatic activities of the rumen microbiota. Moreover, recently, high sequencing approaches of ruminal bacterial community (Enjalbert et al., 2017) suggest that main BH bacterial species are still unknown.

Previous enzyme kinetics approaches based on *in vitro* cultures of ruminal content were proposed by Troegeler-Meynadier et al. (2006) and Moate et al. (2008). These two studies assumed that the BH reactions responded to Michaelis–Menten kinetics. The rates and efficiencies of the three reactions were calculated in the first study, K_m_ and V_max_ in the second. Both studies used *in vitro* incubations with live ruminal microorganisms, providing an overall estimation of microbial activity due to both enzymes carried out by the inoculum and enzymes produced during bacterial growth. These latter enzymes can fail to strictly reflect enzymes produced *in vivo* due to different fermentation conditions, including pH, which can also affect enzyme activity (Kepler and Tove, 1967).

In this study, an alternative approach was developed to specifically study the enzymes from mixed ruminal bacterial species, using inactivation of bacterium by chloramphenicol, an inhibitor of protein synthesis in prokaryotes (Allison et al., 1962). Using this method, our objective was to estimate kinetics parameters of biohydrogenating enzymes produced *in vivo*. Different experiments were done to estimate classic enzymatic kinetics parameters: average rates and efficiencies of reactions (observation of enzymatic inhibition or saturation), Michaelis–Menten K_m_ and V_max_ (enzyme affinity for substrates, enzymatic mechanism, metabolic role of enzymes in BH), pH and quality of inoculum (variation of enzymatic activities). A first experiment explored the effect of time duration on the evolution of average rates and efficiencies of all BH reactions and studied over time the intermediates formed according to the nature of PUFA. A second one used different concentrations of PUFA to calculate K_m_ and V_max_. A last experiment investigated the effects of the donor cow’s diet, which could affect nature and quantity of enzymes, and the effect of pH, which could affect enzyme activity, on the two of C18:2n-6 BH pathways.

## Materials and Methods

### Preparation of the Enzymatic Solution

Rumen fluid was collected on two consecutive days and mixed from two cannulated dry Holstein cows fed a hay-based diet (56% meadow hay, 18% barley, 18% wheat, 6% soybean meal, as percent of DM), except for Experiment 1.3. To investigate the two C18:2n-6 BH pathways (Experiment 1.3): t11 or t10, rumen fluids were collected 1 day from two dry dairy cows equipped with a ruminal cannula, one of them receiving a high-starch high-fat diet (to provoke a t11 to t10 shift of C18:2n-6 BH), and the other a low-starch low-fat diet, given in two equal meals per day, after 14 days of adaptation as previously described (Zened et al., 2013). Briefly, the two diets were based on corn silage and contained soybean meal and a mineral mixture: the high-starch high-fat diet (33.1% starch and 7.3% crude fat, DM basis) contained 49% of wheat and barley mixture and 5% of sunflower oil, 32% of corn silage and 13% of soybean meal, and the low-starch low-fat diet (21.5% starch and 2.9% crude fat, DM basis) contained 14% of alfalfa hay, 69% of corn silage, and 16% of soybean meal (on a DM basis). All diets are supplemented with minerals and vitamins, and cows had access to water in permanence.

One liter of ruminal fluid was taken from each cow with a vacuum pump in the ventral sac 30 min before the morning meal and strained through a metal sieve (1.6-mm mesh). Rumen fluid was quickly transferred to the laboratory in anaerobic conditions at 39°C. In the laboratory, 200 mg of chloramphenicol were immediately added to 200 mL of rumen fluid in a flask subsequently gassed with CO_2_, closed, and stirred. The dose of 1 mg chloramphenicol/mL of ruminal fluid was adapted from Allison et al. (1962), who used 50 μg/mL to inhibit bacterial growth of *E. coli* in 5 × 10^7^ cell count cultures, when our cultures initially contained around 10^11^ copies of total rumen bacterial species. After 5 min of continuous stirring, the mixture was placed at 39°C during 5 h for a complete inhibition of bacterial growth according to Allison et al. (1962) and Rocha et al. (1996), resulting in an enzymatic solution (see **Supplementary Figures S1**, **S2** for adjustment of chloramphenicol treatment).

### Experimental Designs

#### Experiments 1.1 and 2.1: Rates and Efficiencies of the BH Steps, Isomers Produced

The enzymatic solution (1 mL) was incubated with a bicarbonate buffer solution (pH = 7; 23.89 g/L of Na_2_HPO_4_, 12H_2_O + 9.24 g/L of Na_2_HCO_3_; v:v), in a 20-mL cell culture vial. For the Experiment 1.1 C18:2n-6 and for Experiment 2.1 C18:3n-3 was added in vials at a concentration of 0.5 mg/mL of culture (purity ≥ 99%, Sigma, Co., St. Louis, MO, United States) in order to saturate the isomerase. Filled vials were gassed with CO_2_, placed in a water bath at 39°C and agitated during 1, 2 or 3 h, in four replicates. Non-incubated vials (0 h) were prepared in the same way but were immediately frozen. The experiments were done in parallel, and repeated once the next day.

#### Experiments 1.2 and 2.2: K_m_ and V_max_ of C18:2n-6 and C18:3n-3 Disappearances

The enzymatic solution (2 mL) was incubated with the bicarbonate buffer solution (v:v) described above, in a 20-mL cell culture vial containing 0.0625, 0.125, 0.25, or 0.5 mg/mL of C18:2n-6 for Experiment 1.2 and of 18:3n-3 for Experiment 2.2 (purity ≥ 99%, Sigma). Filled vials were gassed with CO_2_, placed in a waterbath at 39°C and agitated during 1 h, in four replicates for each amount of C18:2n-6 added. Two non-incubated vials per amount of C18:2n-6 added were prepared in a same way for determination of initial concentrations of C18:2n-6, and immediately frozen. Experiments were done separately on to consecutive days: d1 for Experiment 1.2 and d2 for Experiment 2.2, since the estimation of Michaelis–Menten kinetic constants needs fixed quantity of enzyme.

#### Experiment 1.3: Effect of pH on Δ12 and Δ9 Isomerases

The enzymatic solution obtained from the cow receiving the high-starch high-fat diet was referred as HSHF, and that from the cow receiving the low-starch low-fat diet was referred as LSLF. One mL of each enzymatic solution was incubated with 1 mL of a pH 5.5 or 7.0 buffer solution based on bicarbonates and phosphates (Troegeler-Meynadier et al., 2006). The pH 7 buffer was the same that in Experiment 1.1, and the pH 5.5 buffer was a high phosphate buffer (pH = 5.5; 8.72 g/L of KH_2_PO_4_ + 0.93 g/L Na_2_HPO_4_, 12H_2_O + 2.31 g/L of Na_2_HCO_3_). These buffers led to initial pH values around 6.8 and 6.2, respectively, after mixing with enzymatic solutions in vials containing 0.25 mg/L of C18:2n-6 (purity ≥ 99%, Sigma). For each of the four combinations of pH buffer and rumen fluid, four vials were immediately frozen for initial status determination and four vials were gassed with CO_2_, placed in a water bath at 39°C and agitated for 1 h.

No similar experiment was done with C18:3n-3 because its BH pathways are still debated excepted t11 one (Doreau et al., 2016).

For the three experiments, reactions were stopped by placing the vials into ice at the end of incubation time. Then, the vial contents were frozen and lyophilized.

### Chemical Analysis

The FA of all incubated and non-incubated vials were extracted and methylated *in situ* with the procedure of Park and Goins (1994), except that the solution of 14% of borontrifluoride in methanol was replaced by a solution of methanol-acetylchloride (10:1). Nonadecanoic acid (C19:0) was used as the internal standard at a dose of 0.8 mg. The FA methyl esters were then quantified by GC (Agilent 6890N, equipped with a model 7683 auto injector, Network GC System, Palo Alto, CA, United States), with a fused silica capillary column (CPSil88, 100 m × 0.25 mm ID, 0.20 μm film thickness; Chrompack-Varian, Middleburg, Netherlands).

For the analysis of samples, two GC analyses were necessary to separate the FA of interest for this study. For both analysis, flame ionization detector temperature was maintained at 260°C and the injector at 255°C, the split ratio was 1:50, hydrogen was the carrier gas. Then for the first passage on GC, the carrier gas had a constant flow of 1 mL/min. The samples were injected in 1 μl of hexane with an automatic injector. Initial temperature of oven was 60°C, held for 2 min, increased by 10°C/min to 150°C, held at 150°C for 12 min, increased by 2°C/min to 175°C, held at 175°C for 20 min, increased by 5°C/min to a final temperature of 225°C and maintained 10 min. This method allows the separation of C16:1 from C17:0 *anteiso*, and of t-C18:1 except t13+t14 C18:1 which coelutes with oleic acid (c9-C18:1), but it did not allow the separation of C18:3 from C20:1. So, a second method was realized to separate these coeluted FA. For this analysis, the carrier gas had a constant pressure of 160 kPa. The samples were injected in 0.5 μl of hexane with an automatic injector. Initial temperature of oven was 60°C, held for 3 min, increased by 8°C/min to 190°C, held at 190°C for 13 min, increased by 5°C/min to 225°C, held at 225°C for 10 min, increased by 10°C/min to a final temperature of 230°C and maintained 10 min.

Peaks were identified and quantified by comparison with commercial standards (Sigma, St. Louis, MO, United States), except t-C18:1 isomers other than t9- and t11-C18:1, which were identified by order of elution (Juanéda et al., 2007; Kramer et al., 2008).

### Calculations and Statistics

FA were expressed as mg per vial.

Calculations of the sums were done as following:

t-C18:1 = t6+7+8-C18:1 + t9-C18:1 + t10-C18:1 + t11-C18:1 + t12-C18:1 + t13+14-C18:1 + t15-C18:1 + t16-C18:1
CLA = t10,c12-CLA + c9,t11-CLA + t9,t11-CLA
t10 = t10,c12-CLA + t10-C18:1
t11 = c9,t11-CLA + t9,t11-CLA + t11-C18:1

For the Experiments 1.1 and 2.1, we wanted to follow production of BH intermediates over 3 h incubations in order to determine the major PUFA BH pathway. Calculations of the production of BH intermediates (mg/vial) were done by subtracting the final FA amount from its initial amount (Privé et al., 2010).

Then, calculations were done to estimate the rates and the efficiencies of the reactions composing PUFA BH, as previously described by Troegeler-Meynadier et al. (2006).

The global rate of a reaction (v) results from the disappearance rate of the substrate and the production rate of the product in the vial. For example, the rates (mg/L/h) of the three C18:2n-6 BH reactions (v1, v2, v3) were calculated:

v1=([C18:2n−6]i−[C18:2n−6]t)/t

where [C18:2n-6]i and [C18:2n-6]t represented the concentration of C18:2n-6 at the beginning and at the end of the t h incubation, respectively. Incubation duration, t, was 1, 2, or 3 h.

v2=([C18:2n−6]i−[C18:2n−6]t+[CLA]i−[CLA]t)/t

where [CLA]i and [CLA]t represented the concentration of total CLA isomers measured at the beginning and at the end of the t h incubation, respectively.

$$\begin{array}{r} v3=([C18:2n-6]i-[C18:2n-6]t+[CLA]i-[CLA]t+\\ {}[\text{t}-\text{C}18:1]\text{i}-[\text{t}-\text{C}18:1]\text{t})/\text{t} \end{array}$$

where [t-C18:1]i and [t-C18:1]t represented the concentration of total t-C18:1 isomers measured at the beginning and at the end of the t h incubation.

The efficiency of the reaction (E) was estimated by the ratio between the concentration of substrate disappeared during the incubation period (initial concentration – final concentration) and the total concentration of substrate available for the reaction considered during this period (initial concentration). The efficiency of the reaction 1 was:

E1=([C18:2n−6]i−[C18:2n−6]t)/[C18:2n−6]i

For the reactions 2 and 3, the substrate is also the product of the previous reaction, so that initial concentration must be increased by the previous reaction production. For the reaction 2, the efficiency was:

$$\begin{array}{r} \text{E2}=([\text{C}18:2\text{n}-6]\text{i}-[\text{C}18:2\text{n}-6]\text{t}+[\text{CLA}]\text{i}-[\text{CLA}]\text{t})/\\ \left([\text{C}18:2\text{n}-6]\text{i}-[\text{C}18:2\text{n}-6]\text{t}+[\text{CLA}]\text{i}\right) \end{array}$$

The efficiency of the reaction 3 was:

$$\begin{array}{r} \text{E3}=([\text{C}18:2\text{n}-6]\text{i}-[\text{C}18:2\text{n}-6]\text{t}+[\text{CLA}]\text{i}-\\ {}[\text{CLA}]\text{t}+[\text{t}-\text{C}18:1]\text{i}\\ -[\text{t}-\text{C}18:1]\text{t})/([\text{C}18:2\text{n}-6]\text{i}-[\text{C}18:2\text{n}-6]\text{t}+\\ \left[\text{CLA}]\text{i}-[\text{CLA}]\text{t}+[\text{t}-\text{C}18:1]\text{i}\right) \end{array}$$

The same principle was applied for the four reactions composing C18:3n-3 BH, with four rates (v1, v2, v3, and v4) and four efficiencies (E1, E2, E3, and E4).

For Experiments 1.1 and 2.1, the effect of incubation duration was tested as a fixed effect using the General Linear Model of SYSTAT. The model also included day of incubation as a fixed effect. The analysis was followed by the Tukey’s pairwise comparison test. Differences were considered significant at *P* < 0.05.

The isomerization was modeled by simple Michaelis–Menten kinetics, as reported by Moate et al. (2008). The Lineweaver–Burk representation was used to estimate K_m_ and V_max_ using: 1/V = ((K_m_/V_max_) × (1/[S]i)) + 1/V_max_; the linear relationship was computed with a linear regression. These parameters help to characterize enzyme affinity for its substrates, enzyme mechanism, and even its BH metabolic role. Indeed our enzymatic solution was a mixture of isomerases. The accuracy of the values obtained was verified using the Michaelis–Menten equation: V = (V_max_ [S]i)/(K_m_ + [S]i).

For the Experiment 1.3, the effects of donor cow diet and incubation buffer pH on t11 and t10 isomers productions were tested using the General Linear Model of SYSTAT (Version 9, SPSS, Inc., 1998, Chicago, IL, United States). This experiment would underline the importance of donor diet on the nature and quantity of enzymes available in such enzymatic solutions, and the effect of pH on enzyme activities. The model used was:

$$\begin{array}{r} \text{Variable}=\text{mean}+\text{effect of cow’s diet}+\text{effect of buffer pH}\\ +\text{interation}+\text{ε}. \end{array}$$

## Results

### Experiments 1.1 to 1.3: Linoleic Acid Biohydrogenation

The rate of C18:2n-6 isomerization decreased with incubation duration when its efficiency increased (**Table 1**, Experiment 1.1). The rates of the first and second reductions also decreased with incubation duration but their efficiencies were nearly constant. The reaction rates were significantly higher after 1 h compared to the other durations. The results obtained with 1 h incubations are reported in **Figure 1**.

**Table 1:** Rates (v, mg/L/h) and efficiencies (E, %) of the reactions of BH of 1 mg of C18:2n-6 or C18:3n-3 after 1, 2, and 3 h incubations in an enzymatic solution obtained from cow’s ruminal content (mean ± SD, Experiments 1.1 and 2.1).

		Time of incubation (h)			*P*
		1	2	3	Incubation time
**C18:2n-6**					
- Isomerization	v1	198 ± 13^a^	121 ± 9^b^	94 ± 12^b^	<0.01
	E1	37 ± 2^a^	45 ± 4^ab^	53 ± 7^b^	0.04
- Reduction 1	v2	169 ± 10^a^	102 ± 12^b^	78 ± 13^b^	<0.01
	E2	85 ± 1	84 ± 4	82 ± 5	NS
- Reduction 2	v3	148 ± 10^a^	86 ± 11^b^	64 ± 10^b^	<0.01
	E3	70 ± 2	70 ± 7	69 ± 3	NS
**C18:3n-3**					
- Isomerization	v1	295 ± 26^a^	151 ± 23^b^	130 ± 2^b^	<0.01
	E1	60 ± 5^a^	62 ± 9^a^	80 ± 1^b^	0.02
- Reduction 1	v2	270 ± 39^a^	131 ± 17^b^	124 ± 9^b^	<0.01
	E2	91 ± 6	87 ± 12	96 ± 7	NS
- Reduction 2	v3	163 ± 51^a^	42 ± 12^b^	49 ± 29^b^	<0.01
	E3	58 ± 10	32 ± 11	38 ± 22	NS
- Reduction 3	v4	153 ± 47^a^	32 ± 6^b^	39 ± 30^b^	<0.01
	E4	58 ± 6	35 ± 2	42 ± 22	NS

*^ab^Values with different superscript in a same raw, significantly differ (P < 0.05).*

**FIGURE 2:**
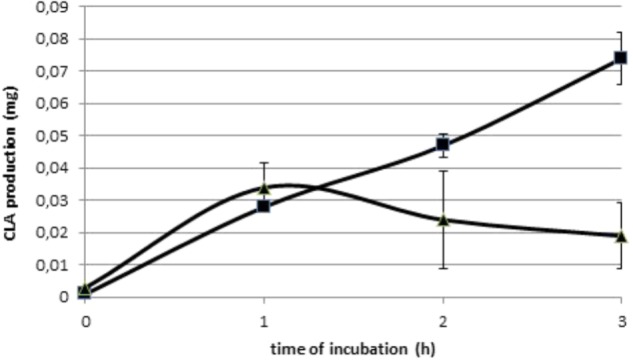
Production (mg) of the two main conjugated linoleic acids: t10,c12-CLA (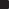) and c9,t11-CLA (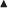), over the 3 h incubations with 1 mg of C18:2n-6 in an enzymatic solution obtained from a cow’s ruminal content (Experiment 1.1).

Initial amounts of C18:2n-6 and C18:3n-3 were 1.07 and 0.04 mg per vial, respectively, in Experiment 1.1. Amount of C18:2n-6 decreased significantly (*P* < 0.01) with duration of incubation until 0.51 mg per vial after 3 h incubation, when C18:3n-3 was constant. Production of all BH intermediates increased throughout incubation (**Supplementary Table S1**), excepted the production of c9,t11-CLA. Its production peaked at 0.034 mg after1 h of incubation (**Figure 2**). **Table 2** only reports the production after 3 h incubation. The t10,c12-CLA and c9,t11-CLA were the most abundant CLA isomers produced and the t10-C18:1 and t11-C18:1 were the most abundant t-C18:1 isomers.

**Table 2:** Amount (mg) of biohydrogenation intermediates produced after 3 h incubation with 1 mg of C18:2n-6 or C18:3n-3 in an enzymatic solution obtained from a cow’s ruminal content (mean ± SD, Experiments 1.1 and 2.1).

	C18:2n-6	C18:3n-3
c11-C18:1	0.001 ± 0.000	0.002 ± 0.001
c12-C18:1	**0.005** ± 0.001	0.000 ± 0.000
c15-C18:1	-0.001 ± 0.000	**0.005** ± 0.007
t4-C18:1	0.000 ± 0.000	0.000 ± 0.000
t5-C18:1	0.000 ± 0.000	0.000 ± 0.001
t6t7t8-C18:1	0.001 ± 0.001	0.003 ± 0.002
t9-C18:1	0.000 ± 0.001	0.002 ± 0.001
t10-C18:1	**0.029** ± 0.014	0.004 ± 0.002
t11-C18:1	**0.059** ± 0.010	**0.065** ± 0.016
t12-C18:1	-0.002 ± 0.000	-0.001 ± 0.000
t13t14-C18:1	0.000 ± 0.000	**0.020** ± 0.008
t15-C18:1	0.000 ± 0.000	0.001 ± 0.002
t16-C18:1	0.000 ± 0.000	0.002 ± 0.000
t10,c12-CLA^∗^	**0.073** ± 0.008	0.008 ± 0.004
c9,c11-CLA	0.000 ± 0.000	0.000 ± 0.000
c9,t11-CLA	**0.016** ± 0.010	0.004 ± 0.004
t9,t11-CLA	**0.008** ± 0.000	0.000 ± 0.001
t11,t13-CLA	0.000 ± 0.000	**0.026** ± 0.006
t11,c15-C18:2	0.000 ± 0.000	**0.450** ± 0.138
c9,t11,c15-CLnA^∗^	0.000 ± 0.000	**0.035** ± 0.023

*Values in bold are those significantly (P < 0.05) different between 0 and 3 h of incubation.*^∗^*CLA, conjugated linoleic acid; CLnA, conjugated linolenic acid.*

In Experiment 1.2, K_m_ and V_max_ for the C18:2n-6 isomerization were 2.0 × 10^-3^ M and 0.6 mM/h, respectively [linear regression of 1/V = ((K_m_/V_max_) × (1/[C18:2n-6])) + 1/V_max_; *P* < 0.01 and *r* = 0.954]. For C18:2n-6 ≥ 1 mM, the raw data did not fit with Michaelis–Menten equation.

**FIGURE 3:**
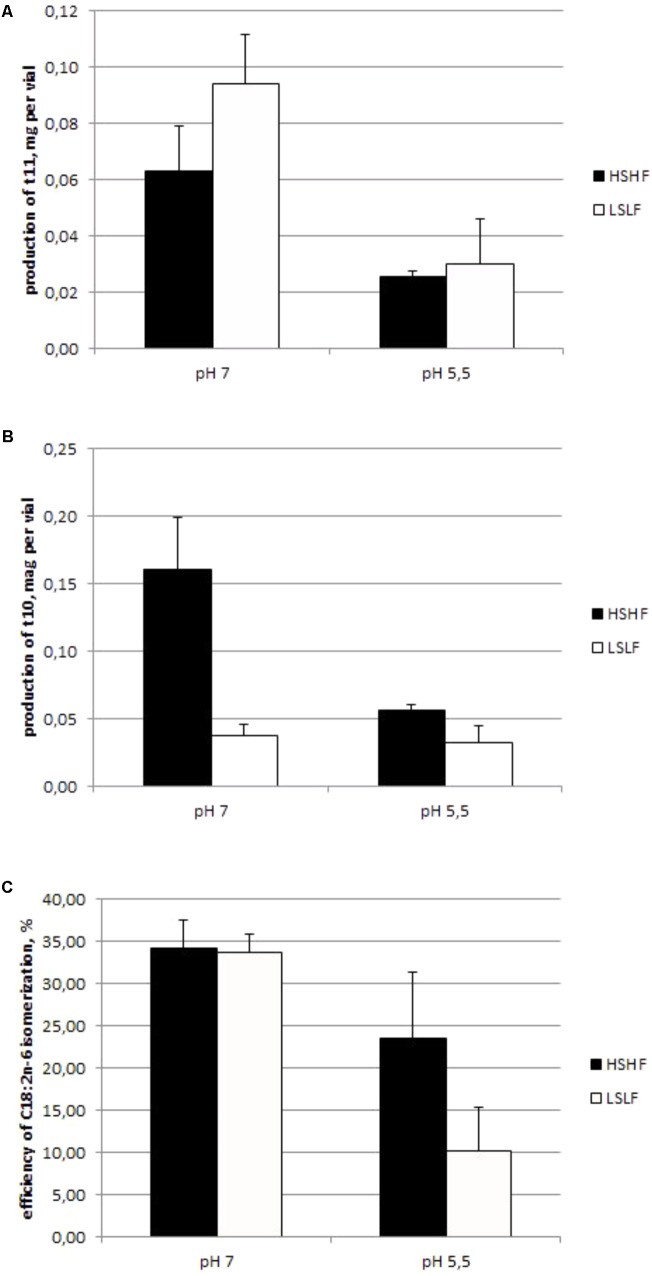
Production (mg) of t11 **(A)** and t10 **(B)** isomers, and C18:2n-6 isomerization efficiency (**C**, %) in a chloramphenicol-treated ruminal content of a cow fed either with a HSHF or a low fat low starch diet (FSLF) and incubated with a high (7.0) or a low (5.5) pH buffer (mean ± SD, Experiment 1.3).

Efficiency of isomerization was negatively affected by low incubation pH (*P* < 0.01; Experiment 1.3; **Figure 3**): 34% with pH 7.0 buffer vs. 17% with pH 5.5 buffer, on average. The effect of diet was only significant (*P* < 0.01) with pH 5.5 buffer: 23% (for HSHF diet) and 10% (for LSLF diet). The pH buffer effect on the production of t10 and t11 isomers is reported in **Figure 3**. A 5.5 pH buffer decreased by around 60% the production of both t10 and t11 isomers, compared to a 7.0 pH buffer (*P* < 0.01). The diet of the donor cow only affected the t10 isomers production (*P* < 0.01), LSLF diet resulting in the lowest values.

### Experiments 2.1 and 2.2: α-Linolenic Acid Biohydrogenation

The rates of the reactions composing C18:3n-3 BH decreased numerically with incubation duration but the difference was only significant after 1 h incubation compared to the others (**Table 1**, Experiment 2.1). Efficiency of the isomerization significantly increased only after 3 h incubation. Efficiencies of the three reductions were nearly constant over time. Results obtained with 1 h incubations are reported in **Figure 1**.

Initial amounts of C18:2n-6 and C18:3n-3 were 0.11 and 0.97 mg, respectively, and significantly decreased over time of incubations until 0.08 and 0.19 mg, respectively after 3 h of incubation. Production of all BH intermediates increased throughout incubation except that of c9,t11,c15-CLnA (**Supplementary Table S2**). Its production peaked at 0.081 mg after 2 h of incubation. **Table 2** only reports the production after 3 h incubation. The CLnA produced was c9,t11,c15-CLnA, and c11,t15-C18:2 was the most abundant intermediate. The t11,t13-CLA was the most abundant CLA isomer produced and the t11-C18:1 and t13+t14-C18:1 were the most abundant t-C18:1 isomers.

In Experiment 2.2, K_m_ and V_max_ for the C18:3n-3 isomerization were 4.3 × 10^-3^ M and V_max_ = 3.4 mM/h [linear regression of 1/V = ((K_m_/V_max_) × (1/[C18:3n-3])) + 1/V_max_ /V: *P* < 0.001 and *r* = 0.993]. For C18:3n-3, all raw data fited with Michaelis–Menten equation.

## Discussion

The BH of C18:2n-6 (**Figure 1**) can follow two major pathways, beginning by either a Δ12 or a Δ9 isomerization, producing c9,t11-CLA and t10,c12-CLA, respectively (Jouany et al., 2007; Zened et al., 2011). The main C18:2n-6 BH intermediates observed in this study (**Table 2**) are in agreement with this general knowledge.

In spite of lower efficiencies than further reactions, isomerization was not the limiting-step of BH, since its rates were higher than those of both reductions, whatever the duration of incubation (**Table 1**). The rate limiting-step was clearly the third reaction, as previously shown by Harfoot et al. (1973). Such a low isomerization efficiency could be explained by a saturation of the reaction by the substrate, as reported before in *in vitro* cultures (Troegeler-Meynadier et al., 2006). Efficiency was the lowest for 1 h incubation, suggesting that the steady state was rapidly reached, which is in agreement with the Michaelis–Menten approximation. Such hypothesis is also supported by the similarity of the 1 h isomerization rate (0.7 mM/h) and V_max_ value (0.6 mM/h). The reductions would be in a dynamic state, with a rate depending on the substrate concentration, as suggested by their constant efficiencies over time when rates decreased.

The rates of all reaction decreased with incubation duration, due to a lack of substrate or a lack of enzymes. After 3 h of incubation the amount of C18:2n-6 was 0.51 mg when 1.07 mg was present at the beginning of the experimentation. *In vitro* with live rumen bacterial species and pH 7 buffer, the fraction of C18:2n-6 escaping BH was estimated at 5.5% of initial C18:2n-6 content (Troegeler-Meynadier et al., 2003), suggesting that a drop of enzyme availability in the present study would cause the drop of reaction rates. It suggests that enzymatic kinetics studies have to be done with no more than 1 h incubation.

Our 2.0 × 10^-3^ M K_m_ for C18:2n-6 isomerization was higher than the 1.2 × 10^-5^ M obtained by Kepler and Tove (1967), but was similar to the 1.6 × 10^-3^ M value obtained by Moate et al. (2008). The first study (Kepler and Tove, 1967) used *B. fibrisolvens* strain A-38 to purify isomerase and worked with this concentrated enzyme solution. The second study (Moate et al., 2008) used whole ruminal content (containing both growing bacteria and enzymes) as in the present study. The difference of enzyme origin (pure enzyme vs. mixture) and assay conditions (solution vs. feed particles and liquid of whole rumen content), between these two previous experiments could explain the different K_m_. Our K_m_ was near the concentration of C18:2n-6 estimated in the rumen of cows receiving the control diet in Zened et al. (2011): 2.7 × 10^-3^ M (113 g of C18:2n-6 for 150 L (rumen content of an adult cow), suggesting an important role of the reaction in ruminal C18:2n-6 metabolism under physiologic conditions. Indeed, in biological systems the K_m_ is often numerically close to the physiological concentration of its substrate.

Our V_max_ of C18:2n-6 isomerization (0.6 mM/h) was very low compared with the 8.6 mM/h V_max_ obtained by Moate et al. (2008) using *in vitro* results of Noble et al. (1974), with 7-h incubations of live ruminal bacterial species. With bacterial growth, the V_max_ represents the ability of bacterium to isomerise C18:2n-6, but with the chloramphenicol-treated inoculum used in our experiment the V_max_ represents the ability of isomerases contained in the inoculum to isomerise C18:2n-6, without any new production of isomerase. The t11 isomers, produced by the Δ12 isomerase, are usually the major intermediates of C18:2n-6 BH (Enjalbert et al., 2017). Our inactivated enzymatic solution had no possibility to synthetize isomerases, in particular Δ12 isomerase, during the incubation, contrary to the *in vitro* cultures used by Moate et al. (2008), and this phenomenon was exacerbated by the inability of the Δ12 isomerase to recycle like a normal enzyme to catalyze more substrate (Kim et al., 2000), inducing a much lower V_max_ in our study than in the study of Moate et al. (2008). Therefore, the inability of the Δ12 isomerase to recycle is a limit of an isomerization study and also explains the decrease of isomerization rate reported above. Moreover, Michaelis–Menten model supposes that there is equilibrium between enzyme (E) + substrate (S) and the complex enzyme-substrate (ES): E + S ES → E + P, with P representing product. If Δ12 isomerase is unable to recycle, the Michaelis–Menten model is not adapted, that’s probably why the model did not match with our raw data when C18:2n-6 ≥ 1 mM. As we did not measure enzyme we cannot conclude about this point. Then, the mismatch of this model for C18:2n-6 isomerization study could also result from the substrate and bacteria localization, i.e., on the solid particles, when Michaelis–Menten equation is applicable for free solution substrate and enzyme. In other words, the tendency of the substrate to bind to feed particles confounds accurate determination of kinetic constants, because the concentrations of added substrate (used for construction of the Michaelis–Menten plots) do not reflect the concentrations of substrate available to the enzyme.

In our study the c9,t11-CLA production was maximal at about 1 h contrary to t10,c12-CLA, which regularly increased during incubation, becoming more abundant than c9,t11-CLA from 2 h of incubation (**Figure 2**). Such difference of kinetics between CLA intermediates of C18:2n-6 BH was similar to that reported *in vitro* with living ruminal microorganisms (Harfoot et al., 1973; Jouany et al., 2007). It could be hypothesized that Δ12 isomerase acted more quickly than Δ9 isomerase, which is consistent with the hypothesis of 2 different mechanisms of action between these two enzymes (Wallace et al., 2007), and with the high affinity of linoleate isomerase of *B. fibrisolvens* for C18:2n-6 observed by Kepler and Tove (1967). However, Δ12 isomerase could not recycle, leading to a rapid decrease of its capacity, so that the c9t11-CLA produced would be immediately reduced by the second reaction. This phenomenon was exacerbated in our chloramphenicol treated cultures, as reported above, so that Δ9 isomerase would become the main BH pathway, resulting in a great production of t10 isomers after 3 h incubation.

Whatever the BH pathway (t11 or t10), the isomerization step was inhibited by a low pH since the production of both isomers was lower with 5.5 pH buffer than 7 pH buffer (**Figure 3**). Indeed, Δ12 and Δ9 isomerases have a similar optimal pH around 7.2 (Kepler and Tove, 1967; Deng et al., 2007). In our study, near to this optimal pH, LSLF resulted in a much lower Δ9 isomerization capacity than HSHF diet, underlying a low content of this enzyme, possibly due to a low abundance or activity of the producing bacterial species in the rumen of donor cows. The shift from t11 to t10 isomers of C18:2n-6 observed with a low rumen pH (Zened et al., 2013) would therefore be linked to a modification of ruminal bacterial community rather than to a difference of enzyme efficiencies. Nevertheless for any origin of rumen content and ruminal pH, when there was few C18:2n-6 in the media the t11 were the dominant isomers (Zened et al., 2012), suggesting that t10 appeared when the t11 way was unable to saturate PUFA because of a lack of producing bacterium and a very low Δ12 isomerase activity, and that Δ12 isomerase had a higher affinity for C18:2n-6 than Δ9 isomerase. This adaptation of the ecosystem could be due to the potential toxicity of PUFA for rumen bacterial species (Maia et al., 2007). Nevertheless we cannot conclude about the reason of this compensation but it seems to be essential for ruminal ecosystem.

The BH of C18:3n-3 (**Figure 1**) produced mainly t11,c15-C18:2, the main CLA was t11,t13-CLA, and the main t-C18:1 were t11-C18:1 and t13+14-C18:1 (**Table 2**), as previously described (Jouany et al., 2007; Honkanen et al., 2016). The t13 isomers would result from the conversion of t11,c15-C18:2 to t11,t13-CLA (Fukuda et al., 2009).

The isomerization of C18:3n-3 to c9,t11,c15-CLnA and the reduction of CLnA to t11,c15-C18:2 were very rapid, contrary to the reduction of t11,c15-C18:2 to t11-C18:1, which is the rate-limiting step of C18:3n-3 BH. The increase over time of t10 isomers concentration could result from the BH of C18:2n-6, because its initial value (0.11 mg) was not negligible. Indeed, the ratios of t10 isomers production/initial C18:2n-6 amount were similar, i.e., 9.5 and 10.9 for Experiments 1.1 and 2.1, respectively. The c9,t11-CLA and a little part of t11-C18:1 probably also resulted from C18:2n-6 BH.

Because of the different possible BH pathways for C18:3n-3 BH (Honkanen et al., 2016), the estimation of the kinetic parameters of C18:3n-3 is very difficult and was rarely reported (Kepler and Tove, 1967). The parameters based on the major pathway, with t11,c15-C18:2 as an intermediate (Honkanen et al., 2016), are reported in **Figure 1**.

Similar to C18:2n-6 isomerase, C18:3n-3 isomerase presented a K_m_ (4.3 × 10^-3^ M) higher than that reported by Kepler and Tove (1967), 2.3 × 10^-5^ M, and probably for the same reasons. This higher K_m_ could result from an adaptation to herbivorous regimen which is usually rich in C18:3n-3. In the 150 L rumen of a cow consuming 12.5 kg of DM of a grass containing around 12 g of C18:3n-3 per kg of DM, the concentration of C18:3n-3 after a meal would be 3.6 × 10^-3^ M, so close to the value of isomerase K_m_.

Compared to C18:2n-6 isomerization, C18:3n-3 isomerization presented a higher K_m_, underlying a lower affinity of our enzymatic solution for C18:3n-3 than C18:2n-6 as reported for the isomerase of *B. fibrisolvens* by Kepler and Tove (1967). V_max_, v, and E were also higher for C18:3n-3 isomerization than for C18:2n-6 one, suggesting a higher efficiency of the C18:3n-3 isomerization. This discrepancy between a lower affinity and a higher efficiency could be explained by the great capacity of C18:2n-6 to saturate its own isomerization reaction, contrary to C18:3n-3. Indeed, Kepler and Tove (1967) reported that isomerization is inhibited by C18:2n-6 from 50 μM, against 100 μM for C18:3n-3. In the same way, Troegeler-Meynadier et al. (2003) did not observe a saturation of C18:3n-3 isomerization when its concentration increased, contrary to C18:2n-6. That was probably the case in the present study since the 1 h incubation rate (1.1 mM/h) was lower than V_max_ (3.4 mM/L). All 18:3n-3 BH reactions would be in dynamic states. Moreover, the C18:3n-3 kinetic responded well to the Michaelis–Menten model, even if the reaction occurs on solid particles.

Previous experiments devoted to the study or rumen BH enzymes used either purified enzymes from one single bacterium, which can fail to represent the diversity of rumen enzymes, or living mixed bacterial species, which continuously produce new enzymes. By inactivating bacterium with chloramphenicol, the present experiment allowed the study of isomerases produced *in vivo* by mixed ruminal bacterial species, and the main results about enzymatic kinetics are summarized in **Figure 1**. Concerning isomerization of C18:2n-6, this study confirmed the high saturability of isomerization by C18:2n-6, and the inhibition of both t11 and t10 pathways by a low pH. The enzymatic solutions produced t11 isomers faster than t10 isomers, but during a shorter period of time, probably because of the inability of Δ12 isomerase to recycle. All results suggested that when Δ12 isomerization is lacking, Δ9 isomerization must then do the BH of PUFA. This could also explain why, in spite of a higher affinity for the isomerase, C18:2n-6 has a lower efficiency to be isomerized than C18:3n-3. This is probably an adaption of herbivorous to regimen rich in C18:3n-3. The rate-limiting step of both C18:2n-6 and C18:3n-3 BH was the second reduction after a very efficient first reduction. Then, the Michaelis–Menten model would be not adequate for C18:2n-6 isomerization study when it matched for C18:3n-3 isomerization. Another approach needs to be developing for the exploration of C18:2n-6 BH, separating t10 and t11 pathways.

## Data Availability

All datasets generated for this study are included in the manuscript and the supplementary files.

## Ethics Statement

Cows were reared according to the European Directive (2010/63/EU) and cannulation of cows was agreed by an ethical committee (CE “Science et santé animales” registered under number 115 with the Ministry of Research).

## Author Contributions

AM designed the experiment. FE, AZ, and AM drafted the main manuscript text and carried out the statistical analyses, prepared all the figures, and provided critical feedback on content. YF, AM, and AZ performed the FA analyses. M-LC, AM, YF, and AZ performed the preparation of the enzymatic solution and the *in vitro* incubations. All authors reviewed the manuscript and approved the final version.

## Conflict of Interest Statement

The authors declare that the research was conducted in the absence of any commercial or financial relationships that could be construed as a potential conflict of interest.

## Supplementary Material

FIGURE S1 Evolution of protein amounts (mg/vial) in strained (1.6 mm) ruminal fluid cultures treated by chloramphenicol at a dose of 1 mg/mL during 1, 2, 3, 4, and 5 h incubations in 40 mL culture vials at 39°C with a bicarbonate buffer (pH = 7, 15 mL of buffer for 15 mL of mix rumen fluid-chloramphenicol) containing urea (1.85 g/L of buffer) and starch (6 g/L of buffer) to supply N and energy to ruminal bacterial species. After incubation, vials were centrifuged at 21000 *g* during 30 min at 4°C, crude proteins (ISO 5983) were assayed in the pellets.

FIGURE S2 Estimation of the total number of bacterial species by quantitative polymerase chain (qPCR, DNA copies/mL of culture) in 24 h ruminal cultures (100 mL of 1.6 mm trained rumen fluid + 100 mL of bicarbonate buffer + 3 g of wheat bran as substrate) with 0, 100, 200, 300, 400, or 500 mg of chloramphenicol, for an initial value of 1.04 × 10^11^ DNA copies/mL of cultures at 0 h. This trial was repeated three consecutive days to obtain three replicates for each chloramphenicol dose. Assays were performed using the ABI Prism 7900HT sequence detection system (Applied Biosystems, Carlsbad, CA, United States) in optical grade 384-well plates in a final volume of 10 μL. The SYBR Green assay reaction mixture contained template DNA, a specific primer set at 100 nM (forward: ACTCCTACGGGAGGCAGCAG; reverse: ATTACCGCGGCTGCTGG) and 1X of Power SYBRs Green PCR Master Mix (Applied Biosystems, Carlsbad, CA, United States). A dissociation curve was added to SYBR Green assays to check the specificity of the amplification. Then, the results were compared with a standard curve to obtain the number of target copies in the sample. The standard DNA curves were generated by amplification of the serial 10-fold dilutions of a reference plasmid containing the target 16S rRNA gene (Accession No. EF445235 *Prevotella bryantii*).

TABLE S1 Amount (mg) of intermediates of C18:2n-6 biohydrogenation according to incubation duration (Experiment 1.1).

TABLE S2 Amount (mg) of intermediates of C18:3n-3 biohydrogenation according to incubation duration (Experiment 2.1).

## Abbreviations

BH: biohydrogenation
CLA: conjugated linoleic acid
CLnA: conjugated linolenic acid
DM: dry matter
FA: fatty acid
GC: gas chromatography
HSHF: high starch high fat diet
LSLF: low starch low fat diet
PUFA: polyunsaturated fatty acid
v: rate of the reaction
E: efficiency of the reaction
[S]: substrate concentration
