# Supplementary figures and images for: Enzymatic Study of Linoleic and Alpha-Linolenic Acids Biohydrogenation by Chloramphenicol-Treated Mixed Rumen Bacterial Species
Source: Front Microbiol. 2018 Jul 3;9:1452. doi: 10.3389/fmicb.2018.01452 (PMC6037716; doi:10.3389/fmicb.2018.01452)

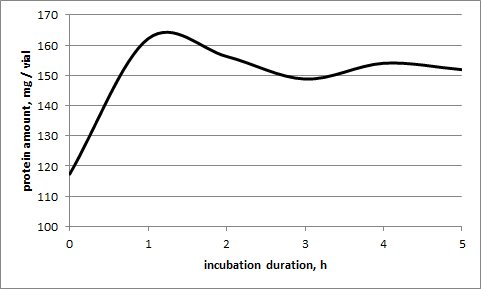

Supplement: FIGURE S1 — Evolution of protein amounts (mg/vial) in strained (1.6 mm) ruminal fluid cultures treated by chloramphenicol at a dose of 1 mg/mL during 1, 2, 3, 4, and 5 h incubations in 40 mL culture vials at 39°C with a bicarbonate buffer (pH = 7, 15 mL of buffer for 15 mL of mix rumen fluid-chloramphenicol) containing urea (1.85 g/L of buffer) and starch (6 g/L of buffer) to supply N and energy to ruminal bacterial species. After incubation, vials were centrifuged at 21000 g during 30 min at 4°C, crude proteins (ISO 5983) were assayed in the pellets. [file Image_1.JPEG]

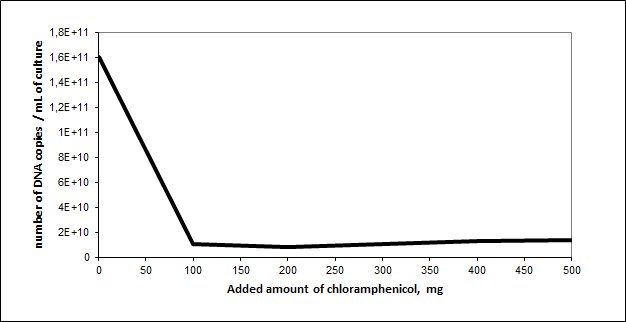

Supplement: FIGURE S2 — Estimation of the total number of bacterial species by quantitative polymerase chain (qPCR, DNA copies/mL of culture) in 24 h ruminal cultures (100 mL of 1.6 mm trained rumen fluid + 100 mL of bicarbonate buffer + 3 g of wheat bran as substrate) with 0, 100, 200, 300, 400, or 500 mg of chloramphenicol, for an initial value of 1.04 × 10^11^ DNA copies/mL of cultures at 0 h. This trial was repeated three consecutive days to obtain three replicates for each chloramphenicol dose. Assays were performed using the ABI Prism 7900HT sequence detection system (Applied Biosystems, Carlsbad, CA, United States) in optical grade 384-well plates in a final volume of 10 μL. The SYBR Green assay reaction mixture contained template DNA, a specific primer set at 100 nM (forward: ACTCCTACGGGAGGCAGCAG; reverse: ATTACCGCGGCTGCTGG) and 1X of Power SYBRs Green PCR Master Mix (Applied Biosystems, Carlsbad, CA, United States). A dissociation curve was added to SYBR Green assays to check the specificity of the amplification. Then, the results were compared with a standard curve to obtain the number of target copies in the sample. The standard DNA curves were generated by amplification of the serial 10-fold dilutions of a reference plasmid containing the target 16S rRNA gene (Accession No. EF445235 Prevotella bryantii). [file Image_2.JPEG]
