# Supplementary material for: Enzymatic Study of Linoleic and Alpha-Linolenic Acids Biohydrogenation by Chloramphenicol-Treated Mixed Rumen Bacterial Species
Source: Front Microbiol. 2018 Jul 3;9:1452. doi: 10.3389/fmicb.2018.01452 (PMC6037716; doi:10.3389/fmicb.2018.01452)
Supplement: TABLE S1 — Amount (mg) of intermediates of C18:2n-6 biohydrogenation according to incubation duration (Experiment 1.1). [file Table_1.DOCX]

**Table S1.** Amount (mg) of intermediates of C18:2n-6 biohydrogenation according to incubation duration (Experiments 1.1).

|  |  | | | | SEM | P* |
| --- | --- | --- | --- | --- | --- | --- |
|  | 0 | 1 | 2 | 3 |  |  |
| C18:0 | 0.567^a^ | 0.581^a^ | 0.584^a^ | 0.615^b^ | 0.007 | 0.006 |
| c11-C18:1 | 0.005 | 0.005 | 0.005 | 0.006 | 0.001 | NS |
| c12-C18:1 | 0.002^a^ | 0.004 | 0.006 | 0.007^b^ | 0.001 | **0.031** |
| c15-C18:1 | 0.001 | 0.001 | 0 | 0 | 0 | NS |
| t4-C18:1 | 0 | 0 | 0 | 0 | 0 | NS |
| t5-C18:1 | 0 | 0 | 0 | 0 | 0 | NS |
| t6t7t8-C18:1 | 0.004^a^ | 0.004 | 0.005^b^ | 0.005 | 0 | **0.013** |
| t9-C18:1 | 0.002 | 0.002 | 0.002 | 0.002 | 0 | NS |
| t10-C18:1 | 0.003^a^ | 0.009 | 0.025 | 0.032^b^ | 0.006 | **0.047** |
| t11-C18:1 | 0.056^a^ | 0.087 | 0.105^b^ | 0.115^b^ | 0.007 | **0.002** |
| t12-C18:1 | 0.009 | 0.007 | 0.005 | 0.007 | 0.001 | NS |
| t13t14-C18:1 | 0 | 0 | 0 | 0 | 0 | NS |
| t15-C18:1 | 0.004 | 0.004 | 0.004 | 0.004 | 0 | NS |
| t16-C18:1 | 0.007 | 0.006 | 0.006^a^ | 0.007^b^ | 0 | **0.036** |
| t10,c12-CLA | 0.001^a^ | 0.028^b^ | 0.047^c^ | 0.074^d^ | 0.003 | **<0.001** |
| c9,c11-CLA | 0 | 0 | 0 | 0 | 0 | NS |
| c9,t11-CLA | 0.003^a^ | 0.034^b^ | 0.024 | 0.019 | 0.006 | **0.029** |
| t9,t11-CLA | 0.000^a^ | 0.004^b^ | 0.005^c^ | 0.008^d^ | 0 | **<0.001** |
| C18:2n-6 | 1.074^a^ | 0.749^b^ | 0.592^bc^ | 0.508^c^ | 0.043 | **<0.001** |
| C18:3n-3 | 0.040^a^ | 0.038^ab^ | 0.037^b^ | 0.038^ab^ | 0.001 | **0.025** |

*effect of incubation duration (General Linear Model, SYSTAT).

NS: non significant; CLA: Conjugated Linoelic Acid

^abcd^values with different superscript in a same raw, significantly differ (P<0.05; Tukey test).
