# Supplementary material for: Enzymatic Study of Linoleic and Alpha-Linolenic Acids Biohydrogenation by Chloramphenicol-Treated Mixed Rumen Bacterial Species
Source: Front Microbiol. 2018 Jul 3;9:1452. doi: 10.3389/fmicb.2018.01452 (PMC6037716; doi:10.3389/fmicb.2018.01452)
Supplement: TABLE S2 — Amount (mg) of intermediates of C18:3n-3 biohydrogenation according to incubation duration (Experiment 2.1). [file Table_2.DOCX]

**Table S2.** Amount (mg) of intermediates of C18:3n-3 biohydrogenation according to incubation duration (Experiments 2.1).

|  | 0 | 1 | 2 | 3 | sem | P |
| --- | --- | --- | --- | --- | --- | --- |
| C18:0 | 1.056 | 1.141 | 1.110 | 1.132 | 0.023 | 0.116 |
| c11-C18:1 | 0.011 | 0.012 | 0.012 | 0.013 | 0.000 | 0.079 |
| c12-C18:1 | 0.005 | 0.005 | 0.005 | 0.005 | 0.000 | NS |
| c15-C18:1 | 0.002 | 0.003 | 0.003 | 0.007 | 0.002 | NS |
| t4-C18:1 | 0.000 | 0.000 | 0.000 | 0.000 | 0.000 | NS |
| t5-C18:1 | 0.000 | 0.000 | 0.001 | 0.000 | 0.000 | NS |
| t6t7t8-C18:1 | 0.008 | 0.009 | 0.008 | 0.011 | 0.001 | **0.043** |
| t9-C18:1 | 0.004 | 0.005 | 0.005 | 0.006 | 0.000 | 0.091 |
| t10-C18:1 | 0.007^a^ | 0.008 | 0.008 | 0.011^b^ | 0.000 | **0.029** |
| t11-C18:1 | 0.105^a^ | 0.134 | 0.149^b^ | 0.170^b^ | 0.009 | **0.006** |
| t12-C18:1 | 0.011 | 0.011 | 0.010 | 0.010 | 0.000 | NS |
| t13t14-C18:1 | 0.022^a^ | 0.030 | 0.034 | 0.042^b^ | 0.003 | **0.009** |
| t15-C18:1 | 0.008 | 0.009 | 0.009 | 0.009 | 0.001 | NS |
| t16-C18:1 | 0.012 | 0.013 | 0.013 | 0.014 | 0.000 | 0.059 |
| t10,c12-CLA | 0.000 | 0.011 | 0.011 | 0.008 | 0.003 | NS |
| c9,c11-CLA | 0.003 | 0.003 | 0.003 | 0.003 | 0.000 | NS |
| c9,t11-CLA | 0.000 | 0.002 | 0.005 | 0.004 | 0.002 | NS |
| t9,t11-CLA | 0.008 | 0.009 | 0.008 | 0.008 | 0.000 | NS |
| t11,t13-CLA | 0.001^a^ | 0.010^b^ | 0.012^b^ | 0.027^c^ | 0.002 | <0.001 |
| t11,c15-C18:2 | 0.008^a^ | 0.224^b^ | 0.363^bc^ | 0.458^c^ | 0.047 | **0.001** |
| C18:2n-6 | 0.109^a^ | 0.104 | 0.080^b^ | 0.085 | 0.006 | **0.027** |
| c9,t11,c15-CLnA | 0.000 | 0.049 | 0.081 | 0.035 | 0.029 | NS |
| C18:3n-3 | 0.969^a^ | 0.380^b^ | 0.366^b^ | 0.192^c^ | 0.031 | 0.000 |

*effect of incubation duration (General Linear Model, SYSTAT).

NS: non significant; CLA: Conjugated Linoelic Acid; CLnA: Conjugated Linolenic Acid.

^abc^values with different superscript in a same raw, significantly differ (P<0.05; Tukey test).
